# Highlighting the novel effects of high-intensity interval training on some histopathological and molecular indices in the heart of type 2 diabetic rats

**DOI:** 10.3389/fendo.2023.1175585

**Published:** 2023-05-19

**Authors:** Mohammad Rami, Samane Rahdar, Amirhoseein Ahmadi Hekmatikar, D. Maryama Awang Daud

**Affiliations:** ^1^ Department of Sport Physiology, Faculty of Sport Sciences, Shahid Chamran University of Ahvaz, Ahvaz, Iran; ^2^ Department of Basic Sciences, Histology section, Faculty of Veterinary Medicine, Shahid Chamran University of Ahvaz, Ahvaz, Iran; ^3^ Department of Physical Education and Sport Sciences, Faculty of Humanities, Tarbiat Modares University, Tehran, Iran; ^4^ Health Through Exercise and Active Living (HEAL) Research Unit, Department of Biomedical Sciences, Faculty of Medicine and Health Sciences, University Malaysia Sabah, Kota Kinabalu, Sabah, Malaysia

**Keywords:** high-intensity interval training, diabetic, cardiomyopathy, exercise, physical activity

## Abstract

**Background:**

Type 2 diabetes is one of the most common metabolic diseases in recent years and has become an important risk factor for cardiovascular disorders. The first goal is to reduce type 2 diabetes, and in the case of cardiovascular disease, the second goal is to reduce and manage that disorder.

**Materials and methods:**

The rats were divided into 4 groups: Healthy Control (n=8), Diabetes Control (n=8), Diabetes Training (n=8), and Healthy Training (n=8). The protocol consisted of 8 weeks of High-intensity interval (5 sessions per week), where the training started with 80% of the peak speed in the first week, and 10% was added to this speed every week. To measure the level of B-catenin, c-MYC, GSK3B, and Bcl-2 proteins using the western blot method, cardiac pathological changes were measured using hematoxylin and eosin staining, Masson’s trichrome and PAS staining and apoptosis using the TUNEL method.

**Findings:**

Histological results showed that diabetes causes significant pathological hypertrophy, fibrosis, and severe apoptosis in heart tissue. HIIT training significantly reduced pathological hypertrophy and fibrosis in heart tissue, and the rate of cardiomyocyte apoptosis was greatly reduced. This research showed that diabetes disorder increases the levels of B-catenin and c-Myc proteins and causes a decrease in the expression of GSK3B and Bcl-2 proteins. After eight weeks of HIIT training, the levels of B-catenin and c-Myc proteins decreased significantly, and the levels of GSK3B and Bcl-2 proteins increased.

**Conclusion:**

This study showed that HIIT could be a suitable strategy to reduce cardiomyopathy in type 2 diabetic rats. However, it is suggested that in future studies, researchers should perform different intensities and exercises to promote exercise goals in type 2 diabetic cardiomyopathy.

## Highlights

- Today, T2DM is known as the main factor in cardiovascular diseases, which can lead to cardiac disorders such as fibrosis, apoptosis, and oxidative stress in heart tissue.
- Different solutions and strategies have been proposed to reduce and treat cardiovascular disorders, one of the best “treatments without the cost” of exercise.
- HIIT can lead to the improvement of cardiomyopathy, including the reduction of cardiac myocytes’ hypertrophy and the accumulation of collagen (fibrosis) in the heart’s myocytes and around the arterial vessels.
- Also, HIIT improved the disorder of Purkinje fibers in the heart tissue of type 2 diabetic rats.
- In addition, the reduction of apoptosis after HIIT in type 2 diabetic rats in heart tissue was one of the prominent observations of the present study. Also, the reduction of β-catenin and c-Myc protein levels as a result of HIIT in type 2 diabetic rats indicated the positive effects of this exercise in improving the cardiomyopathy of T2DM.
- HIIT can be a suitable strategy to reduce and manage cardiomyopathy in people with T2DM, and to prove this, more future studies are needed to investigate signaling pathways and cardiac function.

## 1 Introduction

Type 2 diabetes (T2DM) is one of the most common metabolic disorders worldwide, which is caused by a combination of two main factors: defective insulin secretion by pancreatic beta cells and the inability of insulin-sensitive tissues to respond appropriately to insulin (1). According to the International Diabetes Federation (IDF), 9.3% (463 million) of adults worldwide had diabetes in 2019, and this figure is expected to increase to 10.2% (578 million) by 2030 to increase to 10.9 percent (700 million) by 2045 (2). The prevalence of T2DM among adults and young people has caused great concern, and people with T2DM in youth often have a more aggressive clinical course than people with T2 diabetes in adulthood (3).

With the prevalence of T2DM worldwide, its side effects and risks are more visible, and this has caused more concerns (3). One complication traditionally associated with T2DM, the risk of which has increased significantly, is cardiovascular disease (4, 5). Meanwhile, diabetic cardiomyopathy is one of the main and most important complications of T2DM; the pathogenesis and clinical features of diabetic cardiomyopathy has been well studied in the last decade, but effective approaches to prevent and treat this disease are limited (6). Diabetic cardiomyopathy is characterized by adverse structural changes (including cardiac hypertrophy and fibrosis), early diastolic dysfunction, and late systolic dysfunction (6, 7). Diabetic cardiomyopathy occurs because of impaired glucose, and lipid metabolism associated with diabetes, leading to increased oxidative stress and activation of multiple inflammatory pathways that mediate cellular and extracellular damage, pathological remodeling of the heart, and diastolic and systolic dysfunction (6). However, important signaling pathways are involved in cardiomyopathy, and targeting them can effectively treat it. Bcl-2 proteins are composed of anti- and pro-apoptotic members and play a key role in regulating apoptosis in the myocardium (8). Anti-apoptotic proteins have been shown to protect against various cardiac pathologies, while anti-apoptotic proteins contribute to heart disease (8). Previous studies have revealed that canonical Wnt/β-catenin/glycogen synthase kinase three beta (GSK3β) and c-Myc (Myc) pathways are widely engaged in regulating various biological processes and play critical roles in the pathogenesis of diabetic rats with myocardial injury (9–11). The inappropriate activation of the Wnt/β-catenin pathway resulting from β-catenin enhancer nuclear localization is also strongly associated with cardiomyopathy (11, 12). Also, with signaling changes in cardiomyopathy, reactive oxygen species (ROS) caused by metabolic disorders caused by excessive superoxide production by the mitochondrial electron transport chain during hyperglycemia can further worsen the disease (13). However, signaling pathways and oxidative stress changes have caused clinical studies to focus more on discovering therapeutic strategies for cardiomyopathy.

Looking at the history of the past, we can see that physical exercise is one of the effective and inexpensive medicines for treating or managing diseases (14). However, studies have focused specifically on the effect of physical exercise and T2DM treatment (15, 16). Different intensities of physical exercise have been investigated to understand the impact on diabetic patients better, but recently, high-intensity interval training (HIIT) has had a special place among researchers (17). Physical exercise appears to improve cardiovascular health in patients with T2DM, but its effects on cardiac structure and function are unknown (17). However, HIIT is a potential treatment for modulating heart risk, cardiomyopathy, blood pressure, and blood glucose (17), but the reasons still need to be clearly understood. In confirmation of these findings, the study of Chavanelle et al. (2017) showed that HIIT has a more positive effect on blood glucose and heart structure changes in T2DM rats compared to moderate-intensity training (18).

Finally, with the emergence of HIIT as a potential treatment for T2DM and cardiomyopathy, many research gaps can still be filled with further studies. In this study, we are trying to determine whether HIIT positively affects these signaling pathways and the histology of the heart by examining the important signaling pathways in diabetic cardiomyopathy, oxidative stress indicators, and histopathological changes. Therefore, we will better understand the impact of HIIT by examining the signaling pathways.

## 2 Material and methods

### 2.1 Animals

**Figure 1:**
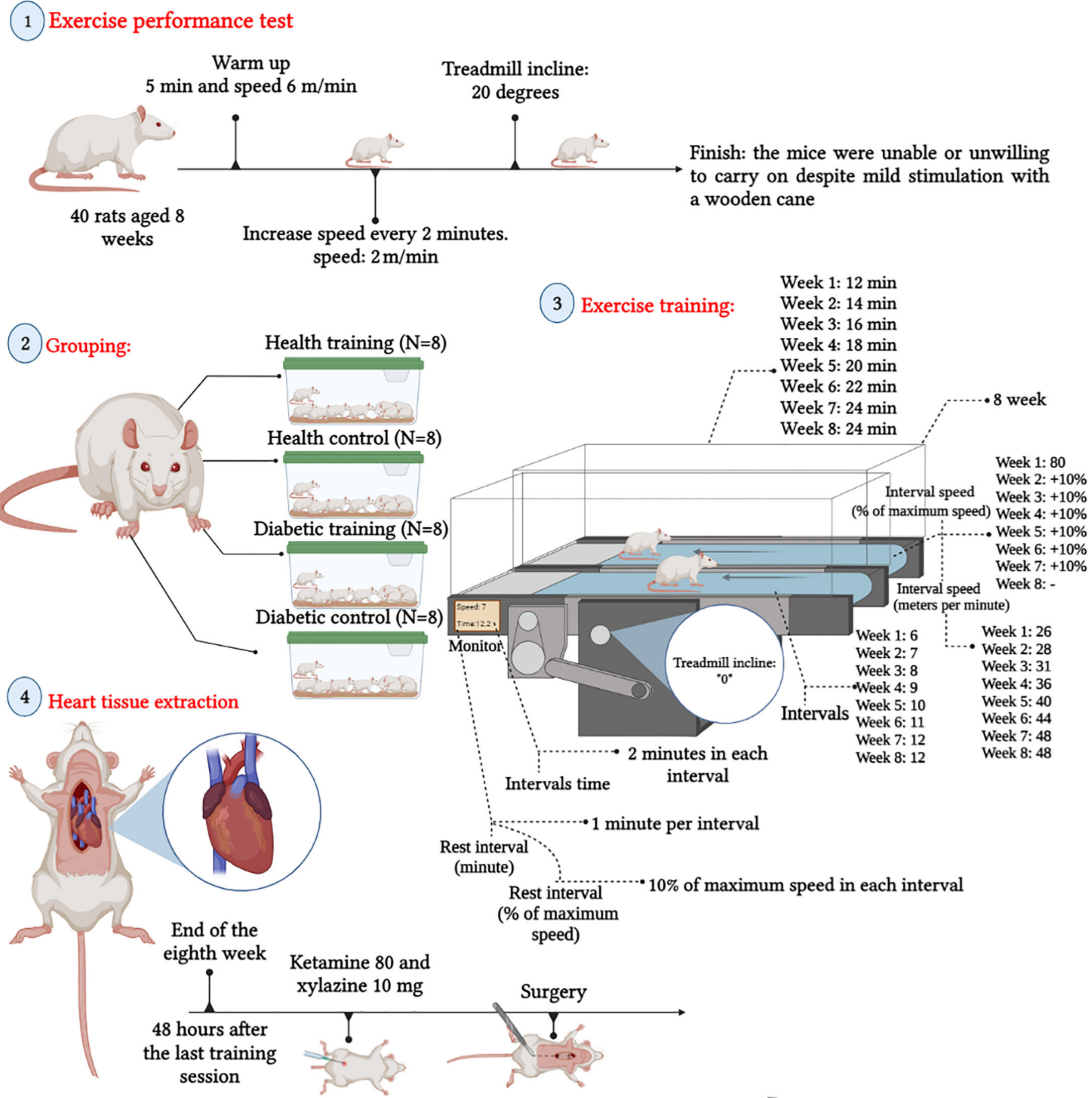
Schematic view training protocol and heart tissue extraction.

We performed all procedures following the Guide for the Care and Use of Laboratory Animals, 8th edition (2011). The local ethics committee approved all procedures (Shahid Chamran University of Ahvaz: EE/1401.2.24.158669/scu.ac.ir). Eight-week-old rats were housed in individual cages with a natural light/dark cycle of 12 h:12 h and fed a standard diet. After one week of adaptation, 32 out of 40 rats were selected for the study after the running test, and the best runners were randomly divided into healthy control (n=8), diabetes control (n=8), healthy training (n=8) and diabetes training (n=8) groups (See **Figure 1**). All rats were dissected simultaneously, and all procedures and experiments were performed for all rats in accordance with each other.

### 2.2 Diabetes induction

Rats in the diabetes groups consumed a high-fat diet (HFD) containing 55% of energy from lard and soybean oil, 31% of carbohydrates, and 14% of protein for two months. The diet of healthy rats was equivalent to 10% fats, such as soybean oil, 76% carbohydrates, and 14% protein (19). After this period, the rats in the diabetes groups fasted for 12 hours. Then a 35 mg/kg dose of Streptozotocin (STZ) solution (Sigma, Germany) was injected intraperitoneally. Two weeks after STZ (Solutions needed to prepare STZ in **Supplementary 1**) injection, the blood glucose of the animals was measured through the tail vein of the rats using a glucometer (Roche Diagnostics K.K., Tokyo, Japan). Animals with fasting blood glucose (FBG) higher than 210 mg/dl were considered diabetic and were included in the study (19). Rats in the control group were injected with an equivalent volume of citrate buffer.

### 2.3 Exercise performance test and exercise training

An exercise performance test was taken from the rats to formulate the training protocol. Before the start, the rats underwent a 2-week familiarization period with the treadmill. Then, Rats were first placed on a treadmill and warmed up for 5 minutes at a 6 m/min speed. Speed was then increased by steps of 2 m/min every 2 min until the rats were unable or unwilling to carry on despite mild stimulation with a wooden cane. In the whole length of the test, the slope was 20 degrees (18). After determining the intensity and speed of the rats, the training protocol began (20). The protocol consisted of 8 weeks of training (5 sessions per week), where the training started with 80% of the peak speed in the first week, and 10% was added to this speed every week. The speed stayed the same in the final two weeks to maintain the adaptations got. The training sessions comprised 6×2-minute training in the first week and continued to 12×2-minute training in the seventh and eighth weeks. After each training, a minute of active rest was performed at a speed of 10 m/min (See **Figure 1**).

### 2.4 Heart tissue extraction

At the end of the eighth week and 48 hours after the last training session, intraperitoneal injection of ketamine 80 and xylazine 10 mg anesthetized the rats. Then, the heart tissue was separated under sterile conditions and immediately transferred to a negative 70 freezer (model 88FD-2-93-A, Iran Madas Company).

### 2.5 Measurement of superoxide dismutase concentration

The measurement of superoxide dismutase (SOD) activity is based on the mechanism of inhibition of Nitrotetrazolium blue (NBT) decline by the xanthine-xanthine oxidase system as a superoxide producer. The optical absorption of each sample was read at 550 nm for 5 min every 30 s. To obtain the percentage of inhibition of NBT reduction by SOD enzyme, the formula corresponding to the Rendox kit was used (Rendox-UK). The activity of the enzyme was obtained by adjusting the percentage of inhibition to the standard curve and reported based on the international unit of protein (µmol/min.g tissue).

### 2.6 Measurement of malondialdehyde concentration

The concentration of Malondialdehyde (MDA) enzyme is based on photometric principles. The basis of this method is the formation of MDA-TBA complex between one molecule of malondialdehyde and two molecules of thiobarbituric acid. Thiobarbituric acid reactive substance (TBARS) was measured from supernatant. In summary, trichloroacetic acid and TBARS reagent were added to the supernatant, then the mixture was placed in an incubator at 100°C for 80 min. Afterwards, it was cooled on ice and centrifuged at RPM 1000 for 2 min. The optical absorption was read at 532nm. The TBARS results were expressed as MDA equivalents using by standard tetraethanoxypropane curve.

### 2.7 Antibodies

β-catenin (β-catenin (E-5): sc-7963, SANTA CRUZ), GSK-3β (GSK-3β (11B9): sc-81462, SANTA CRUZ), c-Myc (c-Myc Antibody (9E10): sc-40, SANTA CRUZ), Bcl-2 (Bcl-2 (C-2): sc-7382, SANTA CRUZ) and HPRT (HPRT Antibody (F-1): sc-376938, SANTA CRUZ).

### 2.8 Protein extraction and western blot analysis

To measure proteins, lysis buffer was first prepared (Preparation of lysis buffer in **Supplementary File 1**). Then, tissue samples were frozen in the -70 freezer to prepare tissue homogenate and western blot test. Also, the Bradford method was used to determine the amount of protein in the tissue homogenate. (Analysis method in **Supplementary File 2**). Then, in the next step, polyacrylamide gel electrophoresis with SDS was used (Procedure, buffers, and solutions required for SDS-PAGE and different steps of polyacrylamide gel electrophoresis with SDS in **Supplementary File 3**). After electrophoresis, blotting, blocking, incubation, and emergence were performed (Buffers and solutions, transfer steps (blotting), method, and blocking action by blocking buffer (blocking) required in **Supplementary File 3**).

Also, Hematoxylin-eosin (H & E) (To examine histological changes in cellular and structure details), TUNEL (Examining apoptotic changes), and Masson’s trichrome and PAS staining (Examination of histopathological changes) were used to check changes (Staining and preparation method in **Supplementary File 4**).

### 2.9 Statistical analysis

Shapiro-Wilk test was used to check the normality of the data, and Levene’s test was used to check the homogeneity of variances. A mixed ANOVA (composite analysis of variance) test was used to investigate changes in glucose and weight in different stages of exercise. In order to check the difference between the averages in the studied variables, a one-way ANOVA test was used, and in the next step, Tukey’s test was used as a *post hoc* test. The significance level was also considered as P<0.05. Data were analyzed using SPSS version 25 software.

## 3 Results

In this research, we investigated the histopathological and histomorphometric changes in the heart tissue of rat T2DM following HIIT. The histological examination of the heart tissue of non-diabetic and diabetic rats following the HIIT was performed using light microscopy. To evaluate the effect of HIIT on the heart, myocardial tissue was stained with hematoxylin and eosin, Masson’s trichrome, and PAS methods.

### 3.1 Changes in average weight and blood glucose

**Figure 2:**
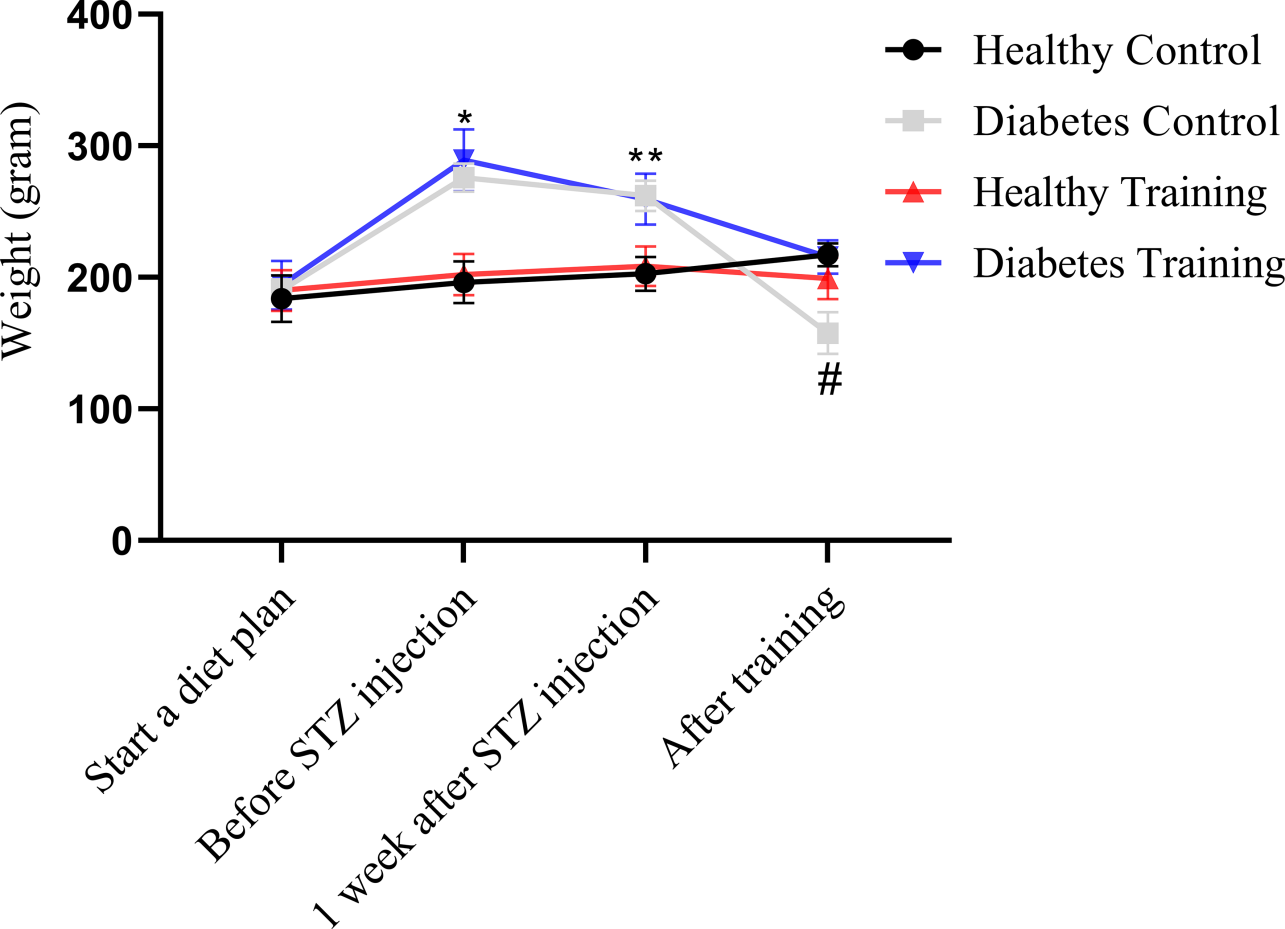
Weight comparison of different groups of rats participating in different stages of the training protocol. *Significant difference between diabetes control and diabetes training groups with healthy control and healthy training groups in the stage before STZ injection; **Significant difference between the diabetes control and diabetes training groups with the healthy control and healthy training groups at the stage of one week after STZ injection; ^#^Significant difference between the diabetes control group and other groups in the phase after the end of the exercise; The significance level is p<0.05.

**Figure 3:**
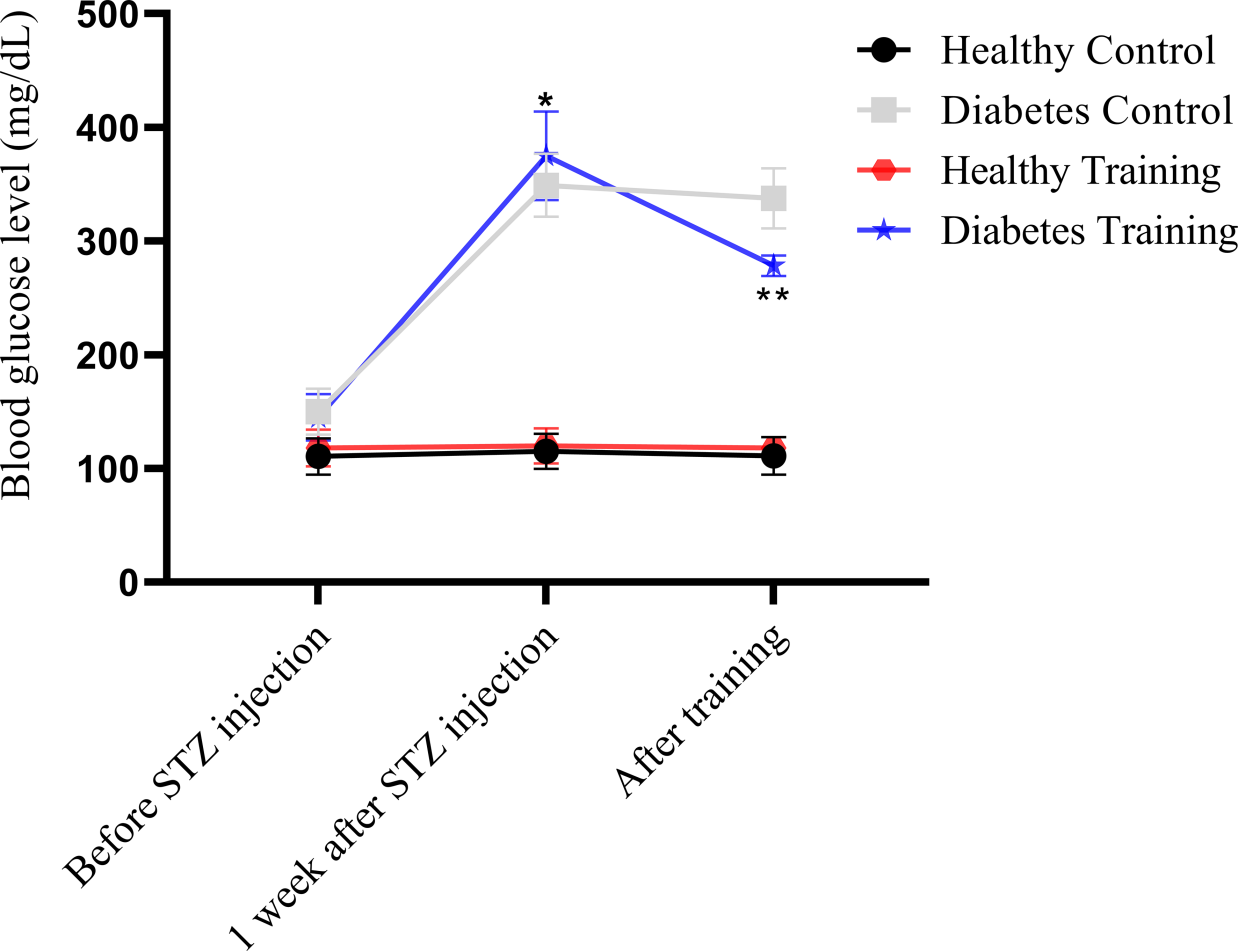
Blood glucose comparison of different groups of rats in different stages of the training protocol. *Significant difference in blood glucose in diabetes control and diabetes training groups with healthy control and healthy training groups at the stage of one week after STZ injection; **Significant difference between diabetes training group and other groups after the end of exercise; The significance level is p<0.05.

**Figures 2**, **3** show the changes in average weight and blood glucose in the weeks and stages of the training protocol. The results of the a mixed ANOVA test showed that the weight of the rats in the diabetes control and diabetes training groups increased significantly from the time of the start of training and high-fat diet to before STZ injection (P<0.001). Also, the weight of this rats showed a significant decrease in the last week and the end of the training protocol (P<0.001). After the end of the training, the weight of the rats in the diabetic control group was significantly lower than all other groups (P<0.001) (see **Figure 2**).

On the other hand, the statistical analysis showed that blood glucose in the diabetes control and diabetes training increased significantly one week after STZ injection (P<0.001). Nevertheless, at the end of the training protocol in the diabetes training group decreased significantly compared to the previous stage (P<0.001) (see **Figure 3**).

### 3.2 Histopathological changes, hypertrophy, Purkinje fibers and collagen area around arterial vessels of heart tissue

**Figure 4:**
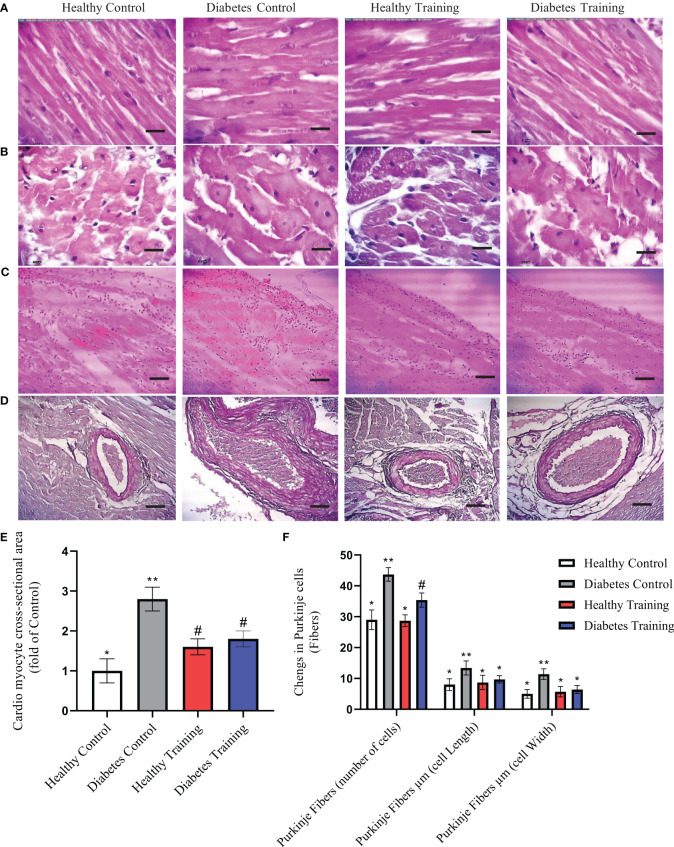
The results of Hematoxylin and Eosin (H&E)-stained cardiac tissue. **(A)** Longitudinal section, **(B)** cross-section, images represent ×40 magnification and scale bars represent 10 μm. **(C)** Purkinje cells (fibers) and **(D)** Collagen area around the arterial vessels, images represent ×10 magnification and scale bars represent 50 μm. **(E)** Diagram of cardio myocyte cross-sectional area. *Significant difference with all groups (P<0.05). **Significant difference with all groups (P<0.001). ^#^Significant difference with diabetic control and healthy control groups (P<0.05). **(F)** Diagram of Purkinje cells (fibers), *Significant difference with diabetes control and diabetes training groups in Purkinje number cells and significant difference with diabetes control groups in Purkinje cell length and Purkinje cell width (P<0.001). **Significant difference with all groups (P<0.001). ^#^Significant difference in diabetes training groups with all groups (P<0.001). Data are expressed as mean ± SEM.

The results of H&E staining showed that the heart tissue of the healthy control group had a normal myofibril structure, which was observed in longitudinal and transverse sections (**Figure 4**, healthy control, A and B) with healthy nuclei located in the canter compared to the diabetes control group. In the diabetes control group, damaged and abnormal myofibrils, faded nuclei, increased collagen connective tissue, and hypertrophy of cardiomyocytes were observed in longitudinal and transverse sections (**Figure 4**, diabetes control, A and B). In the heart tissue of diabetes training rats, the damage of heart tissue myofibrils has decreased, cell nuclei have become clearer, collagen connective tissue has decreased, and hypertrophy caused by diabetes has significantly decreased in longitudinal and transverse sections (**Figure 4**, diabetes training, A and B). Also, this research showed that the average cross-sectional area of myocardial cells in the diabetes control group increased significantly compared to the healthy control group (P<0.001), and pathological hypertrophy was significantly controlled in the diabetes training group after performing the exercise protocol (P<0.05). The healthy training group also showed significant physiological hypertrophy after performing the exercise protocol (P<0.05) (**Figure 4E**).

The results of our study showed that in the diabetes control group, Purkinje fibers similar to cardiomyocyte cells were hypertrophied and their number increased significantly (P<0.001) (**Figure 4C**, diabetes control). HIIT in the diabetes training group significantly reduced the changes caused by diabetes in Purkinje cells (P<0.001) (**Figure 4C**, Diabetes Training). The number, length and width of Purkinje cells were not significantly different in healthy control and training groups (**Figure 4F**).

The results of H&E staining in heart tissue showed that the percentage of perivascular collagen area (PVCA) in the diabetes control group was significantly increased compared to the healthy control group (P<0.001) (**Figures 4D**, diabetes control). Also, the results of this study showed that after a period of HIIT exercise, the percentage of collagen area around the arterial vessels (PVCA) in the diabetes training group showed a significant decrease compared to the diabetic control group (P<0.001) (**Figure 4D**, Diabetes Training). There is no significant difference was observed in the collagen area around the arterial vessels in the healthy training and healthy control groups (**Figure 4D**).

### 3.3 Fibrosis changes of heart tissue

**Figure 5:**
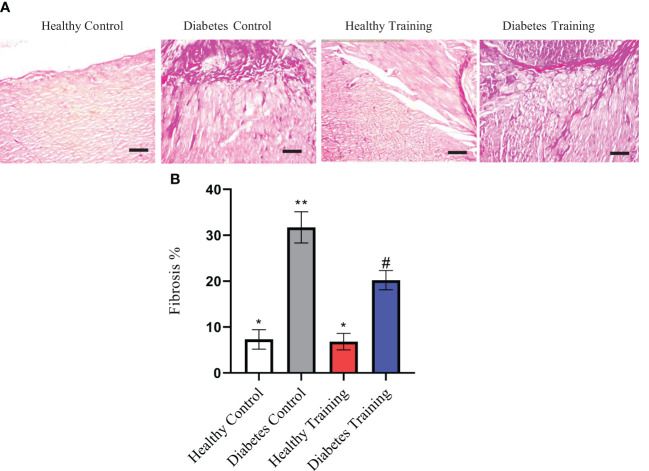
**(A)** The results of PAS staining of rat heart tissue in diabetic and non-diabetic groups following HIIT exercises to investigate the area of cardiac tissue fibrosis. **(B)** Diagram of cardiac tissue fibrosis. *Significant difference with diabetes control and diabetes training groups (P<0.001). All images represent ×10 magnification and scale bars represent 50 μm. **Significant difference with all groups (P<0.001). ^#^Significant difference with all groups (P<0.05). Data are expressed as mean ± SEM.

**Figure 6:**
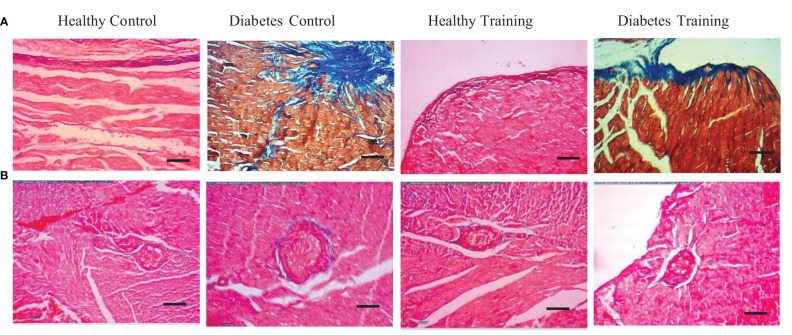
**(A)** The results of Masson’s trichrome staining of rat heart tissue in diabetic and non-diabetic groups following HIIT exercises to investigate the area of cardiac tissue fibrosis. **(B)** Collagen area around the arterial vessels. All images represent ×10 magnification and scale bars represent 50 μm.

In the present study, we showed the area of cardiac tissue fibrosis by examining the extracellular matrix and collagen accumulation using PAS and Masson’s trichrome staining. These areas are visible in the form of collagen scaffolds. This extracellular matrix and collagen accumulation can be seen in purple-red PAS staining and blue in Masson’s trichrome staining (**Figures 5A**, **6A**). The results of PAS and Masson’s trichrome staining of the heart tissue of rats in diabetes groups show the presence of fibrosis in the heart tissue in the diabetes control group, which is obviously larger than the healthy control group (P<0.001) (**Figures 5A**, **6A**). This issue supports the existence of diabetic cardiomyopathy in diabetic model rats with HFD diet combined with STZ injection. The results showed that a period of HIIT improved this pathological abnormality in diabetic hearts with a significant reduction in the area of cardiac tissue fibrosis in the diabetes training group (P<0.05) (**Figures 5A**, **6A**). Normal amounts of collagen tissue can be seen in the healthy control and healthy training groups (**Figures 5A**, **6A**). In addition, the results of Masson’s trichrome staining in heart tissue showed that the percentage of perivascular collagen area (PVCA) in the diabetes control group was significantly increased compared to the healthy control group (P<0.001) (**Figure 6B**). Also, the results of this study showed that after a period of HIIT, the percentage of collagen area around the arterial vessels (PVCA) in the diabetes training group showed a significant decrease compared to the diabetes control group (P<0.001) (**Figure 6B**). The diagram of fibrosis changes in different groups is shown in **Figure 5B**.

### 3.4 Apoptosis rate of heart tissue

**Figure 7:**
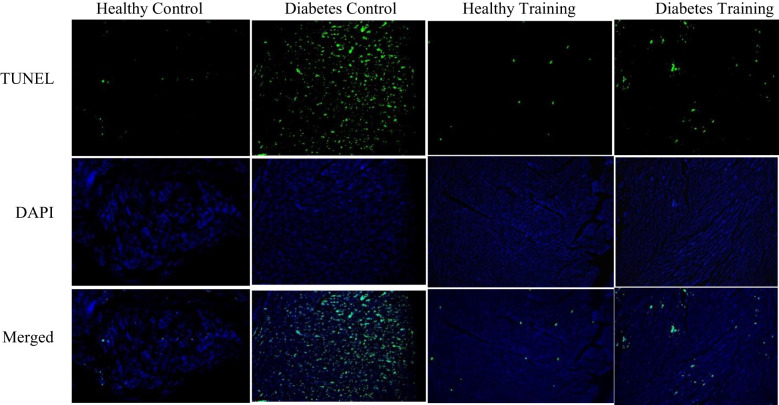
Investigating the apoptosis values of cardiac myocytes of rats in diabetic and non-diabetic groups following HIIT. Green dots show TUNEL positive cells. Fluorescent microscope images represent ×200 magnification.

The results of the present research showed that TUNEL positive cells (apoptotic) were significantly more in the diabetes control group than in the healthy control group (P<0.001) (**Figure 7**). Also, the results of our research showed that the amount of TUNEL positive cells in the diabetes training group showed a significant decrease after eight weeks of HIIT activity (P<0.001) (**Figure 7**). There is no significant difference was observed in the TUNEL positive cells in the healthy control and healthy training groups (**Figure 7**).

### 3.5 Changes in the amount of proteins using the western blot method

**Figure 8:**
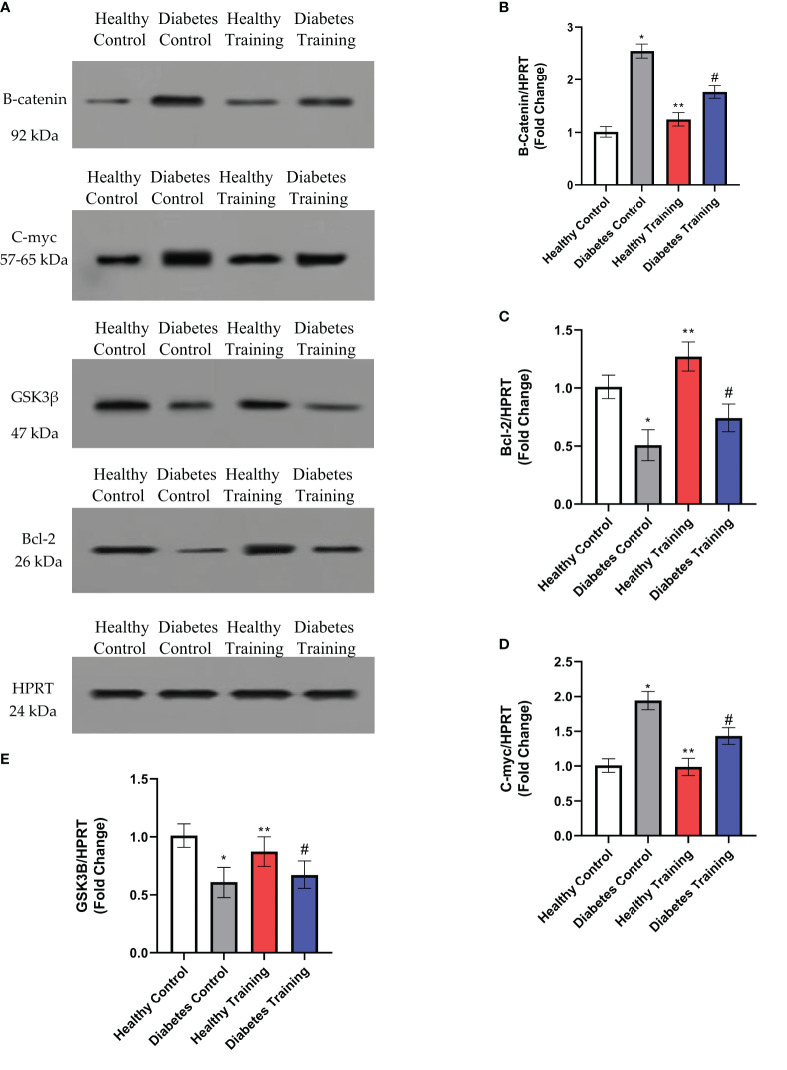
Evaluation of proteins content using western blot method. **(A)** The expression of B-catenin, C-myc, GSK3b and Bcl-2 proteins in the heart tissue of rats in different groups. **(B–E)** The relative ratio of proteins to HPRT levels were determined by image J. *Significant difference in Diabetes Control group with Healthy Control group (P<0.001). **Significant difference in Healthy Training group with all groups in Bcl-2 and GSK3β proteins and significant difference with Diabetes Control and Diabetes Training in B-catenin and C-myc proteins (P<0.05). #Significant difference in Diabetes Training group with all groups in B-catenin, C-myc and Bcl-2 proteins and significant difference with Health Control and Healthy Training in GSK3β protein (P<0.05). Data are expressed as mean ± SEM.

The amounts of B-catenin, GSK3β, C-myc and Bcl-2 proteins in the heart tissue of rats in different groups are shown in **Figure 8**. The results showed that the content of B-catenin and C-myc proteins increased significantly in the diabetes control group compared to the healthy control group (P<0.001). This difference was also significant between the diabetes control and diabetes training groups (P<0.05), which shows that the amount of these two proteins in the diabetes training group has decreased significantly after HIIT. In addition, the content of GSK3β protein in the diabetes control group significantly decreased compared to the healthy control group (P<0.001), while the difference in the content of this protein in the diabetes training group compared to diabetes control group was not significant (P>0.05). The results of the present research also showed that the content of Bcl-2 protein in the diabetes control group was significantly reduced compared to the healthy control group (P<0.001), while in the diabetes training group, the content of this protein was significantly increased than the diabetes control group (P<0.05), the increase in protein content in the healthy training group compared to the healthy control group was also significant (P<0.05).

### 3.6 Antioxidant changes and oxidative stress in heart tissue

**Figure 9:**
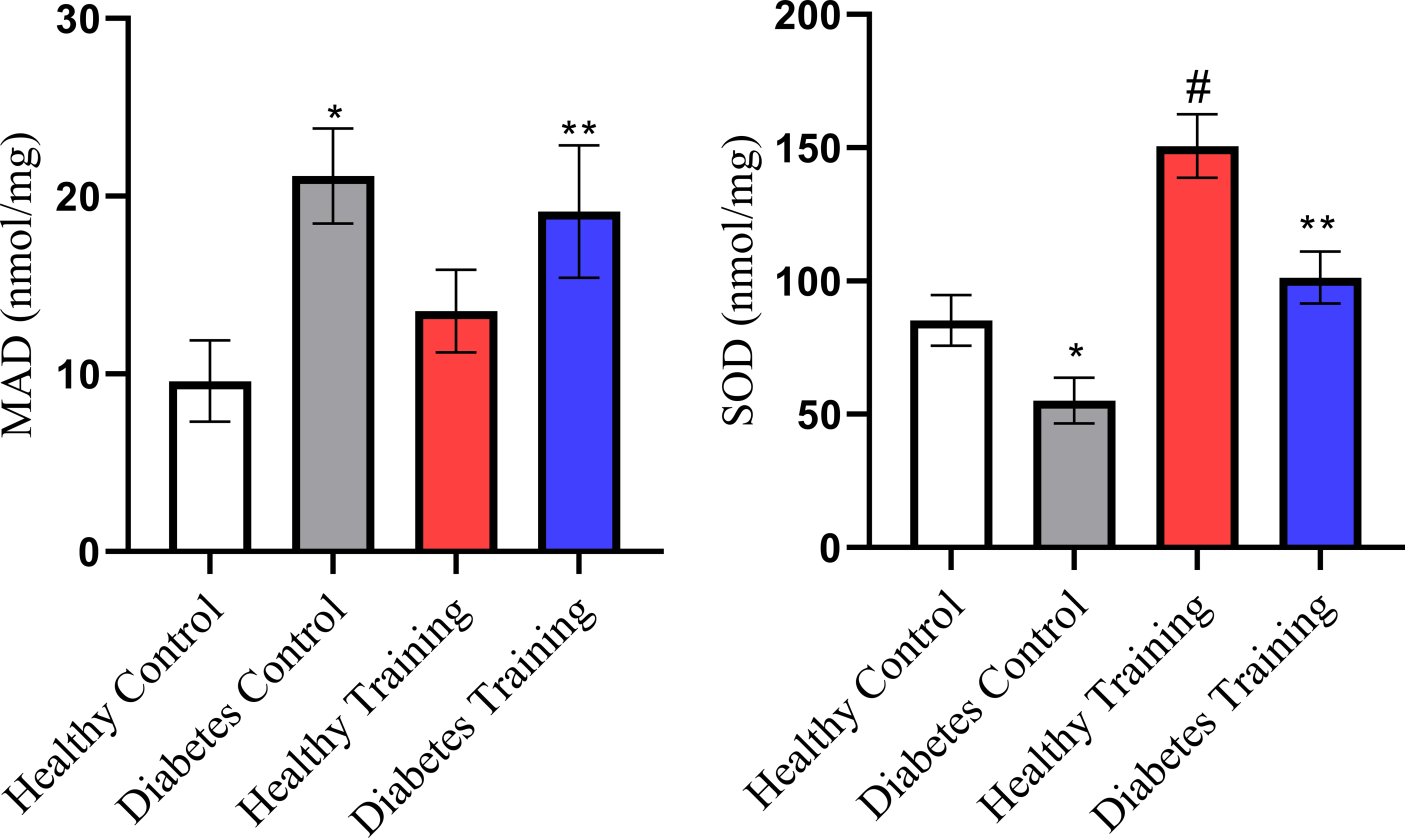
SOD and MDA changes in different groups. *Significant difference in Diabetes Control group with all groups (P<0.001). ^#^Significant difference in Healthy Training group with all groups (P<0.001). **Significant difference in Diabetes Training group with Diabetes Control group (P<0.05). Data are expressed as mean ± SEM.

The results of the current research showed that the concentration of superoxide dismutase (SOD) as an antioxidant index in the diabetes control group was significantly reduced compared to the healthy control group, and in the diabetes training group, the concentration of SOD was significantly increased compared to the diabetes control group (P<0.05). The increase in SOD concentration in the healthy training group was also significant compared to the healthy control group (P<0.001). The results of our study also showed that the concentration of malondialdehyde (MDA) as an index of oxidative stress increased significantly in the diabetes control group compared to the healthy control group (P<0.001). The reduction in MDA concentration in the diabetes training group compared to the diabetes control group was also significant (P<0.05) (**Figure 9**).

## 4 Discussion

In the present study, the results of blood glucose measurements showed that a period of HIIT reduces blood glucose levels in diabetic rats. In confirmation of these results, Cassidy et al. (21) also showed that 12 weeks of HIIT led to blood glucose control in people with T2DM by reducing HbA1c compared to the control group (21). Adams’ study (2013) also showed that in T2DM patients, a 2-week HIIT program increased GLUT4 protein, a marker of insulin sensitivity, and decreased mean blood glucose 48-72 hours after exercise (22).

The results of our research showed that diabetes increases the cross-sectional area of cardiomyocytes in the heart tissue of rats. In line with the results of this research, Novoa et al. in 2017 (23), Wang et al. in 2019 (24), and Lu et al. in 2021 (25) stated that diabetes causes an increase in the cross-sectional area and thickness of cardiomyocytes in rats. Enlargement of the heart in response to stress, hypertension, myocardial injury, or neurohumoral overactivation is associated with cardiac dysfunction and is described as pathological hypertrophy (26).Cardiac function is initially preserved in pressure overload-induced cardiac hypertrophy, described as the adaptive phase; however, our study showed that HIIT significantly reduces the cross-sectional area of cardiomyocytes in the heart tissue of rats with T2DM. Our results are consistent with the studies of Yuan et al., 2020 and Nova et al., 2017 is in line with the report that HIIT can reduce pathological hypertrophy in the heart of T2DM rats (23, 27).

Our study showed that HIIT has significant positive effects on the regeneration of cardiac capacity in T2DM rats and can significantly reduce the fibrosis of the heart tissue of diabetic rats and the collagen area around the arterial vessels. Besides reducing the hypertrophy of cardiac myocytes, it reduces the accumulation of collagen (fibrosis) in the cardiac myocytes and around the arterial vessels. These results are consistent with the findings of Novoa et al. in 2017 (23), Chengji Ji et al. 2019 (28), and Marchini et al., 2020 (29), Lu et al., 2021 (25). The presence of interstitial fibrosis and fibrosis around arterial vessels is a common complication in diabetes and is usually considered an important factor in reducing contractility in the later stages (30). Previous studies reported a decrease in contractility and relaxation and an increase in left ventricular stiffness in STZ-treated diabetic rats. In diabetic models, hyperglycemia disrupts the dynamic balance of collagen synthesis and degradation in the myocardial extracellular matrix by promoting type I and III collagen deposition through converting cardiac fibroblasts to myofibroblasts (29). These results are in line with our observations in the diabetic group, which led to an increase in the level of cardiac tissue fibrosis in diabetic rats.

The results of our study regarding the Purkinje network of heart tissue showed that in the diabetes control group, Purkinje fibers similar to cardiomyocyte cells were hypertrophied, and their number increased significantly. Kang, 2006 states that the normal distribution of Purkinje fibers in the myocardium is proportional to the mass of the heart (31). Cardiac hypertrophy caused by the hypertrophic growth of cardiac myocytes leads to the unbalanced distribution of Purkinje fibers in the regenerating heart (31). Therefore, the conduction of pacemaker potentials becomes problematic. Logantha et al. reported in 2021 (32) that in heart failure, there is extensive remodeling of the Purkinje network at the structural, molecular, and electrical levels, leading to dysfunction and an arrhythmic substrate. Also, therapeutic strategies that prevent heart dilatation may prevent the remodeling of Purkinje fibers and may be beneficial for patients with heart failure. Lanlua et al. also reported in 2012 that cardiac myocytes and Purkinje fibers hypertrophy in the heart of STZ-treated diabetic rats (33). In 2022, Chaturvedi et al. reported that in diabetes, Purkinje cell protein (PCP-4) is degraded by calpain-1, causing contractile dysfunction that can be reduced by exercise (34). Consistent with their results, our study showed that HIIT in diabetic rats significantly reduced diabetes-induced changes in Purkinje cells. However, the effects of different training interventions on the changes of Purkinje fibers seem ambiguous and need more studies.

This research showed that the amount of TUNEL positive cells (apoptotic) cells in the heart tissue of diabetic rats increased significantly. Our study’s results align with the research results of Wu et al., 2017 (35), Xi et al., 2015 (36), Ren et al., 2020 (37), and Nova et al., 2017 (23). Previous studies have shown a close relationship between cardiac dysfunction and myocardial apoptosis and state that controlling myocardial apoptosis is crucial to improving cardiac function (38, 39). The increase in myocardial apoptosis causes the loss of contractile units and cardiac remodeling, which leads to cardiac dysfunction. Studies have shown that, at the cellular level, diabetes increases cardiac myocyte hypertrophy, interstitial fibrosis, and apoptosis (40). This heart damage process causes the production of active oxidative species (ROS) (40). Oxidative stress exists when the production of ROS exceeds its destruction by antioxidant systems (40).

The results of our study also indicate that the values of MDA as an index of oxidative stress in the hearts of diabetic rats increased, and the values of SOD as an antioxidant index decreased in the hearts of diabetic rats. One of the causes of diabetic cardiomyopathy (DCM) is apoptosis, caused by hyperglycemia and abnormal disorder in plasma glucose and insulin level. In fact, long-term hyperglycemia is one of the main causes of ROS formation, insulin resistance, and dysregulation of cytoplasmic calcium. This case makes heart muscle cells susceptible to cell death and ultimately causes changes in muscle contraction and heart failure (41).

Also, our research showed that the amount of TUNEL positive cells and, as a result, the amount of apoptosis in diabetic rats after eight weeks of HIIT showed a significant decrease. Consistent with the results of our study, the results of the study by Chengji and Xiajin (2019) (28), which investigated the effect of two types of low-intensity and HIIT on the apoptosis of heart tissue in diabetic rats, showed that HIIT was more effective in reducing apoptosis than low-intensity training. In contrast, the study by Novoa et al. (2017) (23) showed that, unexpectedly, the level of apoptosis was significantly increased in the diabetic training group; this is when the blood glucose level of their research samples was about 600 mg/dL. While in the diabetes induction protocol, blood glucose levels above 300 mg/dL have been considered as a criterion for diabetic samples, and it seems that they used high doses of alloxan to induce diabetes (42). The data from Novoa et al. (23) study showed that HIIT increases apoptosis in cardiomyocytes of diabetic rats. On the other hand, they showed that a HIIT program had a positive effect on cardiac regeneration, which was evident as a reduction in pathological hypertrophy caused by diabetes and a reduction in collagen deposition (fibrosis) in cardiac tissue, but HIIT increased apoptosis. In the study of Novoa et al. (2017) (23) the training program continued for only four weeks, which may not be the right time to create proper adaptations in intense training. Also, oxidative stress indices increased significantly after exercise in diabetic rats, which can explain the high levels of apoptosis in diabetic rats. On the other hand, it has been shown that HIIT can be an important factor in increased cell death. Based on the evidence, HIIT, besides creating physiological adaptations, may be associated with an ineffective antioxidant defense system of the body and lead to oxidative stress and cell damage (43). Whether HIIT (more than 85% VO2 max) is harmful or beneficial to the heart remains controversial (44). This refers to an increase in energy consumption and a change in the metabolism of substances, which leads to a decrease in the efficiency of ATP synthesis (44). Tissue oxygenation and mitochondrial respiratory chain electron leakage are reduced, easily causing oxidative stress damage. Studies have shown that Wnt/β-catenin signaling is important in regulating cardiac function, but little information is available on its role in DCM (36). It has been shown that Wnt2 participates in early cardiogenesis and is upregulated following cardiac abnormalities (45). As the histopathology results of our study also showed, the DCM process is characterized by pathological hypertrophy of the heart, myocardial remodeling and dysfunction, and myocardial fibrosis, which in severe cases, can lead to heart failure (46). Previous studies have shown that inhibiting NF-κB activation or regulating GSK3β expression may, in turn, limit the transcription of NF-κB downstream factors and the Wnt/β-catenin/GSK3β pathways (47, 48). As a result, it improves the inflammatory response to myocardial damage in diabetic rats. NF-κB and Wnt/β-catenin signaling pathways are known as important regulators in cardiac pathophysiology (12). In the molecular part, our research showed that the amount of β-catenin and C-myc proteins increased significantly in diabetic rats compared to the control group. At the same time, the amounts of GSK3β and Bcl-2 proteins in diabetic rats show a significant decrease compared to the control group (49). C-Myc protein is an oncogenic transcription factor known to regulate cell proliferation, differentiation, and apoptosis as well as cell size (49) and is upregulated during experimental hypertrophy. Since activated Wnt/β-catenin signaling has been observed during DCM development, stabilization and increased levels of β-catenin and its downstream target genes may lead to myocardial injury (36). Stabilized β-catenin facilitates p53-mediated myocardial DNA damage and apoptosis under diabetic oxidative stress through c-Myc upregulation (36). Studies have also shown that systemic inhibition of GSK3β may cause abnormal hypertrophic growth of the heart, possibly leading to heart failure (50). Inhibition of Wnt signaling by activating GSK3β has also reduced the hypertrophic response (50). In another study, Xi et al. (36) found that inhibiting GSK3β stabilizes β-catenin, which may cause cardiac remodeling. Their study stated that the mRNA expression of GSK3β is decreased, and the phosphorylated form of GSK3β (P-GSK3β) is increased in diabetic rats. On the other hand, Al-Damry et al. (51) stated in 2018 that one of the anti-apoptotic mechanisms of AKT is to phosphorylate and thus inactivate GSK3β. They stated that the decreased level of P-GSK3β in diabetic rats reflects the activity of GSK3β. Their study stated that the p-GSK-3β protein level and the p-GSK-3β/GSK-3β ratio were increased in the sitagliptin-treated group, possibly attributed to the restored Akt activity. Al-Damry et al. reported that Akt inhibits GSK-3β activity under normal conditions by phosphorylation. However, in diabetes, the ability of Akt to phosphorylate GSK-3 is reduced, causing GSK3β to remain active and leading to cardiac apoptosis. In our study, the results show a decrease in GSK3β in diabetic rats, which is associated with widespread apoptosis in the heart tissue of rats. On the other hand, Pelozin et al. (52) reported in 2022 that GSK3β is a negative regulator of cardiac hypertrophy. They stated that endurance training decreased GSK3β and thus increased mTOR levels, which is an important factor in cardiac hypertrophy. Perhaps the reason for these contradictions in previous studies and the results of our research is the method used to induce diabetes and the high doses of STZ they used and not using high-fat diets and then injecting a low dose of STZ (42). It is also possible that HIIT, despite the significant increase in GSK3β levels, activating other signaling pathways such as IGF1/AKT/mTOR, is an important factor in achieving physiological hypertrophy and controlling pathological hypertrophy in our research results. However, other factors probably influence the expression and transcription of GSK3β, which were not investigated in this study and need further investigation in the future. One of the components of apoptosis is the increase of the pro-apoptotic protein Bax and the decrease of the anti-apoptotic protein Bcl-2, which causes heart damage due to exposure to oxidative stress. In 2020, Liu et al. (12) stated that the increase in Bax/Bcl-2 ratio in diabetes increases the apoptosis process through the activity of the P53 protein. In the present study, HIIT intervention has decreased the amount of β-catenin and c-Myc protein in diabetic rats. Studies have shown that β-catenin protein and the Wnt/β-catenin signaling pathway play an important role in the pathogenesis of diabetic cardiomyopathy, and inhibition of β-catenin and Wnt can reduce pathological hypertrophy and myocardial fibrosis. Also, in line with the current study’s findings, Yang and et al. (14) showed in their study in 2017 that physical exercise at different intensities inhibits and reduces the expression of proteins of the Wnt/β-catenin signaling pathway in diabetic rats. Their study stated that inactivating the Wnt/β-catenin signaling pathway reduces fat synthesis, improves lipid metabolism, increases glucose absorption and use, prevents muscle atrophy, and ultimately improves insulin sensitivity. Also, the results of our research showed that the amount of anti-apoptotic protein Bcl-2 increased significantly after HIIT in healthy and diabetic groups. Quindry et al. (53) also pointed to the protective function of endurance training (70% of maximal oxygen consumption) in dealing with myocardial apoptosis in rats after ischemia-reperfusion injury. Consistent with the results of our study, Suri et al. (54) reported in 2021 that Bcl-2 gene expression increased in both young and aged rats following HIIT. These findings showed that physical exercise protects the heart against apoptosis and can be a useful strategy to prevent heart problems in people with diabetes. Physical exercise can partially prevent the release of mitochondrial cytochrome C by reducing ROS and preventing cardiac apoptosis (54).

One of the strengths of the present study is the examination of histological and molecular changes, in which we could show the details of the changes after HIIT. Also, investigating signaling pathways and different colorings was another strength of this study in proving the effectiveness of HIIT.

On the other hand, this study also had limitations and weaknesses. One of the weak points of the present study is the lack of cardiac function tests such as ECG due to the lack of laboratory facilities. Therefore, it is suggested that in future studies, researchers examine the functional changes of the heart using echocardiography.

## 5 Conclusion

Although HIIT has been used in medical rehabilitation, the appropriate exercise protocol for preventing cardiac events is still debated. The various strategies used in previous studies also make it difficult to conduct a cross-comparative analysis among them. The results of our research have depicted only a part of the cellular, molecular and histological interactions in the heart tissue of rats suffering from diabetes. This study is not a decisive, solid, and comprehensive achievement regarding the events caused by exercise and its effects on samples with diabetes. However, what is certain is that investigating the effects of intense sports training on sick and healthy samples requires more research. By identifying other factors involved in the homeostasis of cardiomyocytes, researchers can achieve a non-pharmacological and stable strategy to help patients with diabetes and other diseases.

## Data availability statement

The original contributions presented in the study are included in the article/**Supplementary Material**. Further inquiries can be directed to the corresponding authors.

## Ethics statement

The animal study was reviewed and approved by Shahid Chamran University of Ahvaz: EE/1401.2.24.158669/scu.ac.ir.

## Author contributions

Idea, supervisor: MR, writing and editing: AAH, laboratory analysis, SR and edit writing: DAD. All authors contributed to the article and approved the submitted version.
