# Supplementary material for: Highlighting the novel effects of high-intensity interval training on some histopathological and molecular indices in the heart of type 2 diabetic rats
Source: Front Endocrinol (Lausanne). 2023 May 19;14:1175585. doi: 10.3389/fendo.2023.1175585 (PMC10235768; doi:10.3389/fendo.2023.1175585)
Supplement: Supplementary file 1 [file DataSheet_1.docx]

Supplementary Material

Highlighting the novel effects of high-intensity interval training on some histopathological and molecular indices in the heart of type 2 diabetic rats

Mohammad Rami ^1*^, Samane Rahdar^2^, Amir Hossein Ahmadi Hekmatikar ^3^, D. Maryama Awang Daud ^4*^

*** Correspondence:** [M.rami@scu.ac.ir](mailto:M.rami@scu.ac.ir), [dmaryama@ums.edu.my](mailto:dmaryama@ums.edu.my)

# Solutions needed to prepare STZ

## Preparation method of 0.1 M citrate buffer

Dissolve 50 ml of distilled water in 1.47 grams of sodium citrate and adjust its pH to 4.5 with acetic acid.

## How to prepare citric acid

0.51 g of citric acid monohydrate (Sigma, USA) was dissolved in 50 ml of distilled water and kept at room temperature.
