# Supplementary material for: Highlighting the novel effects of high-intensity interval training on some histopathological and molecular indices in the heart of type 2 diabetic rats
Source: Front Endocrinol (Lausanne). 2023 May 19;14:1175585. doi: 10.3389/fendo.2023.1175585 (PMC10235768; doi:10.3389/fendo.2023.1175585)
Supplement: Supplementary file 2 [file DataSheet_2.docx]

Supplementary Material

Highlighting the novel effects of high-intensity interval training on some histopathological and molecular indices in the heart of type 2 diabetic rats

Mohammad Rami ^1*^, Samane Rahdar^2^, Amir Hossein Ahmadi Hekmatikar ^3^, D. Maryama Awang Daud ^4*^

*** Correspondence:** [M.rami@scu.ac.ir](mailto:M.rami@scu.ac.ir), [dmaryama@ums.edu.my](mailto:dmaryama@ums.edu.my)

# Preparation of lysis buffer

To prepare 20 ml of lysing buffer, Tris base 50 mM (0.3 g), sodium chloride 150 mM (0.43 g), triton x100 0.1% (0.02 ml), sodium deoxycholate 25/ 0% (0.05 g), SDS 0.1% (0.02 g) and EDTA (5.84 g) were mixed in 20 ml of distilled water, and its pH was adjusted to 7.4. After adding the mentioned ingredients, using distilled water, the final volume was brought to 50 ml. One protease inhibitor tablet was used for every 10 ml of solution.

# Preparation of tissue homogenate

Tissue samples frozen in -70 freezer were used to prepare tissue homogenate and western blot test. For every 100 mg of tissue, 200 μl of cold lysing buffer was added and the samples were kept at 4°C. The samples were homogenized for 2 minutes at 4°C using a homogenizer (analytikjena, Speed Mill plus, Germany) at 25,000 revolutions per minute (rpm). The samples were centrifuged at 14000 rpm for 10 minutes and the supernatant was transferred to a new microtube. After measuring the protein by the Bradford method, the samples were kept at -70 degrees until the next analysis. To perform SDS-PAGE, the samples were mixed with 2X SDS loading buffer at a ratio of 1:1 and boiled for 5 minutes. After boiling and to eliminate the air vapor created in the microtube, the samples were centrifuged for 5 seconds.

# Protein measurement by Bradford method

This method used BSA bovine serum albumin with a concentration of 1 mg/ml as a standard. For this purpose, to prepare Bradford's reagent, 10 mg of Coomassie blue G-250 was dissolved in 5 ml of 95% ethanol, and 10 ml of 85% phosphoric acid was added to it. Then the volume of the solution was increased to 100 ml. Then, to perform the test, concentrations of 0, 2, 4, 6, 10, 15, and 20 microliters of BSA standard and 20 microliters of tissue homogenous sample were added to the wells of the 96-well plate in two repetitions. 40 μl of Bradford's reagent was added to each well, and piping was done. Then 200 microliters of distilled water were added to each well and kept at room temperature for 5 minutes. The absorbance of the samples was read at a wavelength of 595 nm using a BioTek SX2 (USA) micro-plate reader. The concentration of samples was calculated based on drawing the standard curve of absorbance changes against the concentration of standard samples. By multiplying the number got from the number of the standard curve by 50, the amount of protein in homogenous tissue samples was obtained in terms of ug/ml.
