# Supplementary material for: Highlighting the novel effects of high-intensity interval training on some histopathological and molecular indices in the heart of type 2 diabetic rats
Source: Front Endocrinol (Lausanne). 2023 May 19;14:1175585. doi: 10.3389/fendo.2023.1175585 (PMC10235768; doi:10.3389/fendo.2023.1175585)
Supplement: Supplementary file 3 [file DataSheet_3.docx]

Supplementary Material

Highlighting the novel effects of high-intensity interval training on some histopathological and molecular indices in the heart of type 2 diabetic rats

Mohammad Rami ^1*^, Samane Rahdar^2^, Amir Hossein Ahmadi Hekmatikar ^3^, D. Maryama Awang Daud ^4*^

*** Correspondence:** [M.rami@scu.ac.ir](mailto:M.rami@scu.ac.ir), [dmaryama@ums.edu.my](mailto:dmaryama@ums.edu.my)

# Buffers and solutions required for SDS-PAGE

**30% acrylamide solution:**7.5 grams of acrylamide and 0.2 grams of bis-acrylamide were mixed, and its volume was increased to 25 ml with distilled water. This solution is sensitive to light and was stored in colored glass containers at a temperature of 4 degrees Celsius for a maximum of two months.

**Densifying gel buffer solution, 1 M Tris**: 3 grams of Tris was dissolved in 15 ml of water, and its pH was adjusted to 6.8 with 2 M HCL; Then the final volume reached 25 ml with distilled water.

**Separating gel buffer, 1.5 M Tris**: 4.54 grams of Tris was dissolved in 15 ml of distilled water, and its pH was brought to 8.8 with 1 M HCL; Then the final volume reached 25 ml with distilled water.

**Ammonium persulfate 10%:** 0.1 g of ammonium persulfate was dissolved in 1 ml of distilled water. This solution should be prepared fresh and kept away from light.

10% SDS solution: 2.5 grams of SDS was weighed and made up to 25 ml with distilled water.

**Electrophoresis tank buffer (running buffer):** 3.03 grams of Tris are mixed with 15.15 grams of glycine, and the volume is 900 ml, then 1 gram of SDS is mixed in 10 ml of distilled water, and after dissolving, its volume is brought to 100 ml with distilled water. Finally, we add pre-prepared tris and glycine to 900 ml.

**How to prepare sample buffer (X2) for SDS-PAGE**: To prepare this buffer, 4 ml of distilled water, 2 ml of 0.5 M Tris with pH = 6.8, 1 ml of beta-mercaptoethanol, 5 mg of bromo phenol blue, 1 ml of glycerol and finally 2 ml of 10% SDS were mixed.

**Preparation of SDS-PAGE separating gel (lower gel(:**Table 1 was used to make 12% SDS-PAGE separating gel.

Table 1. Preparation of SDS-PAGE separating gel (lower gel).

| Gel percentage | distilled water (ml) | 30% acrylamide solution (ml) | 1.5 Tris solution (ml) | SDS 10% (ml) |
| --- | --- | --- | --- | --- |
| 12 | 1.6 | 2.0 | 1.3 | 0.05 |

Before pouring the samples into the molds, 55 microliters of ammonium persulfate and 5 microliters of TEMED were added.

**Preparation of condensing SDS-PAGE gel (upper gel)**: Table 2 was used to make SDS-PAGE condensing gel.

Table 2. Preparation of condensing SDS-PAGE gel (upper gel)

| distilled water (ml) | 30% acrylamide solution (ml) | 1 Tris solution (ml) | SDS 10% (ml) |
| --- | --- | --- | --- |
| 1.4 | 0.33 | 0.25 | 0.02 |

Before pouring the samples into the molds, 20 microliters of ammonium persulfate and 2 microliters of TEMED were added.

## Polyacrylamide gel electrophoresis with SDS

Glass molds for SDS-PAGE were first properly washed and degreased with ethanol. Before preparing the gel and pouring it into the glass mold, the two sides of the glass molds were first fixed by clamps, and to insulate the bottom of the mold and prevent the gel from leaking out, the edges of the separators were dipped in paraffin. After adjusting the glass plates and making the lower separating gel, ammonium persulfate and TEMED were added. After mixing, it was slowly poured into the space between the glass plates. Immediately, 2 ml of butanol was slowly poured on the surface of the gel in order to prevent air penetration during the gel's polymerization and smooth its surface. After gel polymerization, butanol was washed on the gel surface using distilled water. Before pouring the upper condensing gel, ammonium persulfate and TEMED were added, and after mixing, it was slowly poured into the space between the glass plates. Then the separating shoulders were placed between the two windows. Before performing electrophoresis, the protein samples were mixed with x2 sample buffer (Loading Buffer) at a one-to-one ratio based on the concentrations got in the Bradford method and boiled for 5 minutes. In this situation, proteins become linear. Then, to remove the created steam, we centrifuged it for 5 seconds and put it in ice. This will cause the vapors to come down and make the solution uniform. After pouring the samples into the electrophoresis wells, the electric current was first established for 15 minutes with a voltage of V60 and then for an hour with a voltage of 100. In each electrophoresis run, one well was dedicated to the protein marker (Ladder) (Thermo Scientific SuperSignal Molecular Weight Protein, 84785).

**Supplementary Figure 1.**. Different stages of polyacrylamide gel electrophoresis with SDS. POWER SUPPLY device (BIO RAD brand, PAC UNIVERSAL POWER model, USA).

# Solutions required for blotting, blocking, incubation and development

**How to prepare transfer buffer (transfer buffer):** 3.03 grams of tris are mixed with 15.15 grams of glycine, and the volume is 900 ml, then 100 ml of methanol is added.

**How to prepare phosphate buffer saline (PBS):** Phosphate buffered saline (PBS), or washing buffer, is a solution of 137 mM NaCl, 2.7 mM potassium chloride, 4.3 mM disodium phosphate, and 1.4 mM potassium dihydrogen phosphate together. To prepare it, 8 grams of table salt (44.58 MW=g/mol), 0.2 grams of potassium chloride (74.56/56 MW=g/mol), 0.61 grams of disodium phosphate (141/96/96 MW=g/mol) and 19/ 0 grams of potassium dihydrogen phosphate (MW=136.09 g/mol) was added to some water. After adjusting Ph=7.4, the final volume was brought to 1000 ml. This solution was kept at 4°C.

**How to prepare 5% Skim Milk blocking buffer:** To prepare 5% blocking buffer, 0.5 grams of Skim Milk was mixed with 10 cc of distilled water and placed on a shaker to dissolve completely.

**Supplementary Figure 2.** Transfer steps (blotting): a) Blotting tank which contains transfer buffer and sandwich; b) Pictures of a sandwich including electrophoresis gel, nitrocellulose paper, Whatman paper and sponge. The act of blocking-by-blocking buffer (blocking): a) separating the paper from the gel and transferring it into the Petri dish; b) immersing the paper with 5% blocking buffer.

## Blotting, blocking, incubation and emergence

After protein electrophoresis using the SDS-PAGE method, the gel was placed in a transfer buffer for 10-15 minutes (Buffers and solutions, transfer steps (blotting) and blocking action by blocking buffer (blocking) required in Supplementary 3). A sandwich comprising five layers, including two layers of sponge, two layers of Whatman paper on both sides, one layer of nitrocellulose paper, and one layer of gel, was prepared. All compounds were immersed in the transfection buffer for 15 min before preparation. After making sure that there were no bubbles in the gel, the sandwich with the cassette base was placed in the blotting tank that was already filled with the transfer buffer so that the blotting paper was placed on the cath and the gel on the anode side. The transmission was done with 60 voltages and for 105 minutes. After the transfer, the paper was washed three times with PBS for 5 minutes each time. Blocking was done by blocking buffer overnight in the refrigerator at 4°C. After blocking, the nitrocellulose paper was washed three times with PBS for 5 minutes each time. After blocking, nitrocellulose paper was used with antibodies diluted 1.2000 to 1.5000 in PBS buffer for one hour at room temperature and incubated on a shaker at 65 rpm. Blotting was done separately for each protein. After incubation, the paper was washed three times with PBS for 5 minutes each time. Incubation of secondary antibodies (mouse anti-rabbit IgG-HRP (sc-2357), SANTA CRUZ) was prepared with 1/2000 dilution in PBS buffer for 1 hour. After incubation, the nitrocellulose paper was washed three times with PBS for 5 minutes each time. Two solutions of the ECL kit (abcam, 133408, USA) were combined in a ratio of 1 to 1 in the amount of 250 microliters and poured onto nitrocellulose paper with the help of a 1000 sampler. We soaked the paper with it for 1 minute. All procedures were performed in a dark room under red light. After leaving the emergency solution, the papers were dried in the environment. Then the papers were placed inside the plastic protective cassette containing the sensitive film, and the bands emerged in the X-RAY processor (LD-14, China). Photosensitive papers were scanned using a JS 2000 scanner (BonninTech, China), and the band density was calculated. To quantify the bands, the density of each protein compared to the calibrator protein in the studied groups compared to the density of the target protein compared to the calibrator protein in the control group was analyzed by JS 2000 software.
