# Supplementary material for: Highlighting the novel effects of high-intensity interval training on some histopathological and molecular indices in the heart of type 2 diabetic rats
Source: Front Endocrinol (Lausanne). 2023 May 19;14:1175585. doi: 10.3389/fendo.2023.1175585 (PMC10235768; doi:10.3389/fendo.2023.1175585)
Supplement: Supplementary file 4 [file DataSheet_4.docx]

Supplementary Material

Highlighting the novel effects of high-intensity interval training on some histopathological and molecular indices in the heart of type 2 diabetic rats

Mohammad Rami ^1*^, Samane Rahdar^2^, Amir Hossein Ahmadi Hekmatikar ^3^, D. Maryama Awang Daud ^4*^

*** Correspondence:** [M.rami@scu.ac.ir](mailto:M.rami@scu.ac.ir), [dmaryama@ums.edu.my](mailto:dmaryama@ums.edu.my)

## Hematoxylin-eosin staining

After heart tissue extraction, three samples from each group were immediately placed in a 10% formalin solution for tissue staining and histological evaluations. After 24 hours, the formalin solution was replaced, and the heart tissue was placed in a fresh 10% formalin solution. Before preparing slides, macroscopic images were taken from the heart using a Dinolite lens. Then, based on the common histology method, slides were prepared from the tissue samples one week after being fixed in formalin. This way, the heart tissue was first dehydrated by ethanol (70% for 24 hours, 90% for 1 hour, and 100% for 1 hour) and then cleaned with gesylol and embedded in paraffin. In the next step, the samples were cut with a microtome device (Leica RM2025, Germany) with a thickness of 5 microns. After going through the usual histological fixation procedures, they were fixed on a glass slide. In this staining, tissue sections were first placed in a dish containing xylol for 5 minutes; then, they were placed in decreasing concentrations of alcohol in each dish for 2 minutes. After that, they were immersed in distilled water for 2 minutes. At this stage, the samples were placed in hematoxylin dye for 15 minutes and then washed with running water for 5 minutes. In the next step, the samples were fixed in eosin for 4 minutes and then washed for 5 minutes. Finally, tissue sections were placed in containers containing ethyl alcohol at this stage with increasing concentrations of 70, 80, 90, 100, and 100 each for 2 minutes. Finally, the samples were placed in two containers containing Gezilol for 20 minutes. In order to preserve the cut and keep it fixed on the slide, they were covered with a slide after staining. For this purpose, lamella and Canada Balsam glue were used. In the last step, stained glass slides were studied under a light microscope using Dinocapture software and Dino-Eye Microscope AM-423X lens (ANMO, Taiwan).

## TUNEL staining procedure

The TUNEL assay, which detects fragmented DNA at the site of the cell nucleus, assessed apoptosis. According to the instructions, this assay was performed using the apoptosis detection kit (TUNEL assay kit, Enhanced, E-CK-A334 FITC). Prepared heart slices were incubated with 50 µL of proteinase K for 30 minutes in a moist chamber and then washed with PBS. Then 100 μl of DNase I working solution was added to the slide and incubated at room temperature (25-37°C) for 10-30 minutes. Equilibration buffer (5×) TdT [E-CK-A334A] was diluted with ddH2O to 1× TdT equilibration buffer. Then 100 microliters of 1x TdT working buffer were added to each slide and incubated at room temperature for 30 minutes. A PBS wash follows each step. The fluid surrounding the tissue on the slides was blotted dry with absorbent paper. Then DAPI (a blue fluorescent dye that binds to DNA-rich regions and penetrates the cell at high concentrations) was added and incubated at room temperature for 5 minutes. The sections treated with DNAse I was considered as a positive control. Sections where proteinase K treatment was replaced with PBS, were considered negative control. After the completion of the protocol, the slices were observed with a microscope (Olympus BX50 Fluorescent Microscope) and Microbin 20 digital camera, and TUNEL positive cells were evaluated using Image J software and reported as Fold of Control.

# Solutions required for the preparation of Masson's Trichrome

**How to make iron alum solution**: Iron ammonium sulfate 4 grams and Distilled water 100 ml.

**How to make solution A**: Fuchsin acid 10 ml, Ponce A 90 ml and Glacial acetic acid 1 ml.

**How to make solution B**: Phosphomolybdic 0.5 grams, Phosphotungstic 0.5 grams and Distilled water 100 ml.

**How to make solution C**: Fast Green 2.5 grams, Deionized water 100 ml and Glacial acetic acid 2.5 ml.

**How to make alcoholic hematoxylin**: Hematoxylin 1 gram and 95% ethanol 10 ml.

**How to make 1% glacial acetic acid solution:** Glacial acetic acid 1 ml and Distilled water 100 ml.

## Trichromatic and PAS staining

The steps of deparaffinization using two xylol containers for 5 minutes each and watering the sample using decreasing alcohol percentages (100, 95, 80, 70) for 2 minutes each and then placing in distilled water were done for 1 minute. The tissue sections were placed in the vicinity of the iron alum for 30 minutes at room temperature. Then they were washed in running water for 5 minutes. Then they were stained in hematoxylin for 30 minutes at room temperature. They washed again in running water for 5 minutes. Schiff's periodic acid was used for differential staining. They were washed in running water for 10-20 minutes. They were stained in solution A for 5 minutes. They were washed in distilled water for 5 minutes. The slides were placed in the vicinity of solution B for 10 minutes. They were stained in solution C for 2-5 minutes. They were washed in acetic acid. The samples were dehydrated in 70%, 90% alcohol, and two containers of absolute alcohol. Gazilol did clarification. The stained slides were mounted by placing a drop of Entlan glue and then placing a slide at a 45-degree angle on them and removing the air from under the slide with forceps. After drying, the slides were ready for study. The nucleus is blue or dark blue, collagen and mucus are blue or green, and cytoplasmic elements, keratin, and muscle are red.
